# Representation and Processing of Lexical Tone and Tonal Variants: Evidence from the Mismatch Negativity

**DOI:** 10.1371/journal.pone.0143097

**Published:** 2015-12-01

**Authors:** Xiaoqing Li, Yiya Chen

**Affiliations:** 1 Key Laboratory of Behavioral Science, Institute of Psychology, Chinese Academy of Sciences, Beijing, China; 2 Jiangsu Collaborative Innovation Center for Language Ability, Jiangsu Normal University, Xuzhou, China; 3 Leiden University Center for Linguistics (LUCL) & Leiden Institute for Brain and Cognition (LIBC), Leiden, The Netherlands; University of Jyväskylä, FINLAND

## Abstract

Pronunciation variation is ubiquitous in the speech signal. Different models of lexical representation have been put forward to deal with speech variability, which differ in the level as well as the nature of mental representation. We present the first mismatch negativity (MMN) study investigating the effect of allophonic variation on the mental representation and neural processing of lexical tones. Native speakers of Standard Chinese (SC) participated in an oddball electroencephalography (EEG) experiment. All stimuli have the same segments (*ma*) but different lexical tones: level [T1], rising [T2], and dipping [T3]. In connected speech with a T3T3 sequence, the first T3 may undergo allophonic change and is produced with a rising pitch contour (T3V), similar to the lexical T2 pitch contour. Four oddball conditions were constructed (T1/T3, T3/T1, T2/T3, T3/T2; standard/deviant). All four conditions elicited MMN effects, with the T1–T3 pair eliciting comparable MMNs, but the T2–T3 pair asymmetrical MMN effects. There were significantly greater and earlier MMN effects in the T2/T3 condition than that in the reversed T3/T2 condition. Furthermore, the T3/T2 condition showed more rightward MMN effects than the T2/T3 condition and the T1–T3 pair. Such asymmetries suggest co-activation of long-term memory representations of both T3 and T3V when T3 serves as the standard. The acoustic similarity between the activated T3V (by the standard T3) and the incoming deviant stimulus T2 induces acoustic processing of the tonal contrast in the T3/T2 condition, similar to that of within-category lexical tone processing, which is in contrast to the processing of between-category lexical tones observed in the T2/T3, T1/T3, and T3/T1 conditions.

## Introduction

In speech communication, pronunciation variation is ubiquitous. A well-documented phenomenon of contextual variation is assimilation, by which a particular phoneme adopts/adapts to the feature(s) of an adjacent phoneme, such as the production of the underlying segment /n/ in green boat as a surface [m]-like sound. In tonal languages, variation can also occur at the supra-segmental level. That is, a lexical tone assigned to a word or morpheme can be realized with different pitch contours contingent upon adjacent tones and discourse context [1–4]. The question that has received much attention in the literature is how the human brain deals with such variability in the speech signal and what are the neural mechanisms that underlie the representation and processing of speech sounds and their variants.

### Models of Pronunciation Variation in Lexical Representation

Different models have been proposed to deal with pronunciation variation, which differ in the level as well as the nature of variation representation and processing. Here, we limit our attention to how regular contextual variation is incorporated into lexical representation. Three main types of accounts have been proposed: the underspecification account, the multi-variant account, and the inference account. The most representative model for the underspecification account is the featurally underspecified lexicon model (FUL), which claims that each word in the mental lexicon is associated with a highly abstract phonological representation with non-distinctive phonological features unspecified, which consequently enables ready accommodation of pronunciation variants in speech [5–7]. This view has been supported by a series of behavioral and neurophysiological studies, in particular for the underspecification of the [coronal] place of articulation in both perception and production (e.g. [8–14]).

At the other end of the spectrum, the multi-variant account (hereafter MV) assumes that contextual variants are stored in the mental lexicon. The nature of these variants have been proposed to be episodic and detailed [15,16] or abstract with varying degrees of strength in memory, which reflects the listener’s frequency of exposure to each particular variant form [17–20]. For example, it was found that American English listeners exhibited a sensitivity to word-specific frequency in their identification of the onset segment (*b* or *p*) of word-nonword continua (e.g., *pretty*-*bretty*), which were embedded in either a flap or a *t* variant carrier word [17]. This suggests the inclusion of the flap variant of spoken words as a stored lexical representation in the mental lexicon.

While the FUL and MV accounts differ in how much speech variability is abstracted away in the mental lexicon, one thing they have in common is that they both locate pronunciation variation at the level of feature or higher-level lexical representation. Both therefore differ from the context-based inference account (hereafter CI), which assumes a single abstract lexical representation. Within the CI, pronunciation variation is resolved at the pre-lexical level via an inference process based on their context (e.g. [21–25]; see also [26–28] for evidence of feature cue parsing of subphonemic modifications which are important for lexical processing). What is important for this and similar accounts is that speech context is essential for the recognition of pronunciation variants so that inference can be made on-line during speech perception and lexical access.

### Lexical Tone and Tonal Variation

It is worth noting that the aforementioned studies all examined speech variants at the segmental level. In addition to consonants and vowels, many languages of the world (60–70%) use pitch variation, together with other (mostly secondary) acoustic features such as phonation, to convey word meanings, known as tonal languages [29]. Unlike consonants and vowels, lexical tones are considered supra-segmental, and their acoustic attribution extends over the whole syllable or multiple syllables. For example, Standard Chinese has four distinctive lexical tones. Produced in isolation, T1 is a high level tone, T2 a high rising tone, T3 a low dipping tone (often accompanied by creakiness), and T4 a high falling tone. Following the tradition of representing lexical tones numerically along five levels of a speaker’s pitch range (with 1 indicating the lowest level and 5 the highest one) [30], we may transcribe the tones as 55, 25, 214, and 53 respectively. The tone-bearing unit in Standard Chinese is the syllable, which typically is also a morpheme. In connected speech, T3 is realized with a rising pitch contour (hereafter Sandhi Rising or T3V) when immediately followed by another T3. Acoustically speaking, T3V is very similar though slightly different from the lexical rising tone (T2) [31–35]. The question that interests us is the consequence of such allotonic variation on the representation and auditory processing of lexical tones in general.

Zhou & Marslen-Willson [36], to our knowledge, is the first perception study that investigates the representation of context-conditioned allophonic tonal variants. They started with the assumption that T3 changes to T2 before another T3 (following [30,37]). They investigated the representational relationship between T2 and T3 in an auditory-auditory priming lexical decision task. The reaction time results showed that the processing of bisyllabic T3T3 compounds were significantly facilitated by a T3TX prime (where X refers to any of the four tones except for T3), which belongs to the same lexical tonal category but surfaces with a different pitch contour. For the same listeners, T3T3 was also inhibited by a T2TX prime, which belongs to a different lexical tonal category but surfaces with a similar pitch contour, when compared to the condition with control primes which do not share lexical tone or segments. On the other hand, for T2TX targets, T3 (i.e., different tone and different contour) showed a significantly more inhibitory effect than T3T3 (different tone but similar contour) and T2 (the same tone and pitch contour), which in turn showed a significantly more inhibitory effect than primes with no segmental or tonal sharing. Taken together, their results point to the possible representational relationship of T3V = T3 and T3V≠T2, but T2≠T3 and T2 = T3V. However, given their assumption that T3T3 undergoes categorical tone sandhi change into T2T3, they were left with the conundrum of the contradictory results of T2 = T3 but T2≠T2/T3. This challenges the assumption that T3 is changed to T2 when followed by another T3, calling for further studies to clarify the nature of T3 contextual sandhi variant.

The more specific goal of our study was therefore to seek neurophysiological evidence for the mental representation and auditory processing of lexical tones and tonal sandhi variants. In particular, we were interested in whether and how in Standard Chinese, the existence of allophonic tonal variant (i.e. T3V) might affect the way a lexical tone (i.e. T3) is represented and processed in general, as compared to the other lexical tones (i.e. T1 and T2). To this end, we will examine whether the processing of T3 would activate automatically its allophonic variant T3V, even when there is no tone sandhi context. Evidence as such should indicate the long-term representation of T3V (together with the canonical T3) in the mental lexicon.

### Neurophysiological and Neuroimaging Studies of Tone Perception

A few studies have investigated the neural basis underlying lexical tone perception. Those using neuroimaging techniques such as functional magnetic resonance imaging (fMRI) or positron emission tomography (PET) demonstrate that the left hemisphere is involved in between-category functional processing of lexical tone (e.g., [38–41]). However, for lower level acoustic processing of lexical tone, right hemisphere is more often recruited (e.g., [42–46]). For example, in an fMRI study, Zhang et al. [46] found that, relative to between-category tonal variation, within-category tonal variation elicited weaker activation in the left middle temporal gyrus (MTG) and, more importantly, stronger activation in the right middle superior temporal gyrus (STG), reflecting low-level acoustic processing. Consistent with this fMRI study, the electroencephalography (EEG) experiment conducted by [42] reported evidence of early right-lateralized mismatch negativities (MMNs) for lexical tone processing, and put forward a two-stage model in which the acoustic signal of a lexical tone is initially processed in the right hemisphere and then the mapping of its functional information (i.e. tonal category) takes place in the left hemisphere. The EEG study conducted by Xi et al. [45] further found that the MMNs elicited by within-category tonal variation are reduced over the left hemisphere as compared to between-category tonal variation. The EEG study in [45] adds further evidence that MMNs evoked by within-category tonal contrast (e.g., pitch level differences within the lexical Tone1) can be lateralized completely to the right hemisphere. Thus, with some differences in the degree of lateralization, the consensus that has emerged from the existing literature is that the left hemisphere is more sensitive to between-category functional processing of lexical tone while the right hemisphere is more sensitive to low-level acoustic processing of pitch variation within a lexical tone.

The above studies made important contributions to the neural basis associated with the perceptual processing of lexical tones in general. However, it is important to note that thus far, the existing literature, which examines either within-or between-lexical tonal contrasts, sheds little light on the possible effects of contextual tonal variation on the neural processing of lexical tones in general.

Another line of research worth noting is EEG studies which examine how acoustic pitch distances influence lexical tone processing. For example, behavioral results showed that in Standard Chinese, T2 (high rising; 25) and T3 (low dipping; 214) are perceptually more similar and more difficult to discriminate than the pair T3 and T1 (high level; 55) [47]. Chandrasekaran, et al. [48] used synthesized pitch contours over the same segmental syllable to examine the MMN responses to large deviant T1/T3 (standard/deviant) vs. small deviant T2/T3 tonal pairs. Their results showed that, for native Chinese speakers, the MMN elicited by the T1/T3 contrast peaked earlier and had larger amplitude than the MMN elicited by the T2/T3 contrast. What remains unclear is whether the above results can be extended to neural processing of naturally produced lexical tones, where multiple cues, in addition to pitch contours, signal lexical tonal contrasts.

### The Present MMN Study

The main goal of the present study was to investigate the role of tonal variants in the representation and neural processing of lexical tones in general. Specifically, we were interested in whether T3, given its tone sandhi variant T3V, is 1) underspecified, 2) represented as its canonical tonal form, or 3) represented as both the canonical form and its variant T3V? To this end, a passive oddball MMN paradigm was used to tap into the neural processing of lexical tones.

Within the oddball MMN paradigm, infrequent acoustic events (deviant) occasionally occur among frequently repeated sounds (standard). The standard stimulus is generally believed to create a sound representation which corresponds, to a large extent, to the high-level long-term memory traces [49–51] while the deviant stimulus evokes a low-level acoustic representation of the speech perception [52–54]. MMN can thus be used to study not only the low-level sensory or acoustic contrast but also the difference between the surface form, extracted from the deviant, and the underlying representation, activated by the standard.

Over the last decade, there has been ample evidence that MMNs reflect the phonetic and phonological properties of the sound system of the listeners’ native language. For example, Dehaene-Mambretz [55] showed that given the same acoustic distance, a large mismatch negativity was observed only when the deviant crossed a phonemic boundary (and not with a within-category deviants). Näätänen, et al. [49] reported enhanced MMN responses to phonemic prototypes in listeners’ native languages, relative to when the deviant is a non-native sound. Such native-language, phoneme-related MMN response enhancement suggests the existence of language-dependent memory traces of speech sounds, which can serve as the basis for auditory mismatch detection.

More recently, a body of studies has also established that MMNs can be employed as a valuable tool to study the neural correlates of lexical access (see reviews in [56,57]). These studies lend support to the view that word memory traces are strongly connected assemblies of neurons; the speed and magnitude of their activation is linked to the strength of internal connections in a memory circuit, such that words generate stronger MMN responses than acoustically similar pseudo-words [58] and high-frequency words elicit stronger and earlier MMNs relative to low-frequency ones [59].

We extended the paradigm to examine the effect of tone sandhi variants on the representation and neural processing of lexical tones in general, inspired by a series of studies that examined MMN responses to tap into the representation of segmental features and their related allophonic alternations (e.g.,[8,14]). We know that MMNs reflect not only high-level long-term memory (e.g. phonological representation in the mental lexicon) but also low-level sensory contrast (e.g. acoustic pitch contour differences of a lexical tone). Given a set of lexical tones (e.g. T1, T2, and T3), low-level sensory contrasts are expected to exist in all of the possible oddball conditions, since T1, T2 and T3 are distinct lexical tones with different pitch contours and native speakers are able to identify lexical tones (produced in isolation) with near ceiling-level accuracy [60]. To tap into the high-level long-term memory representation with regard to lexical tone and tonal variants, a reversed design was adopted, where standard and deviant stimuli in one condition were reversed as deviant and standard in another condition. In other words, we employed four oddball conditions with two tonal pairs (i.e. T1/T3 vs. T3/T1 and T2/T3 vs. T3/T2; 85%/15%). In this way, the standard-deviant acoustic-phonetic contrast, an important factor determining the MMN responses, was identical in both conditions. The specific question that we addressed is whether upon hearing the T3 produced in isolation, its T3V (which only surfaces in the specific T3T3 sandhi context) is also activated as part of the high-level long-term memory of T3.

Given that the allophonic variant of the lexical Low tone (T3V) has a comparable acoustic distribution to that of the lexical rising tone (T2), T3V and T2 can be perceptually ambiguous in the allophonic T3T3 context (against T2T3). The near-neutralization of T3V and T2 has also been reported to enhance the perceptual similarity of T2 and T3 to native Chinese ears, compared to their greater perceptual distance to American English ears [47,48]. T1 and T3, however, are rather distinct in all contexts. Importantly, our design only involved monosyllabic words, which does not introduce the T3 sandhi context. The interesting question that arises here is whether the auditory input of T3 produced in isolation would nevertheless invoke the representation of the sandhi T3V variant (if it is stored as long-term presentation in the mental lexicon), which would then render the T3 and T2 to be less distinguishable.

A critical comparison of our design is between the MMNs elicited in the T2/T3 condition and that in the reversed T3/T2 condition, as compared to the MMNs due to the contrast between T1 and T3 (see Table 1). In the T1/T3 condition, a conflict always occurs, since the mental representation activated by the standard T1 (lexical T1 with high level pitch contour) would be different from the acoustic low pitch contour information extracted from the deviant T3. A similar conflict exists in the T2/T3 condition, since the mental representation (lexical T2 with rising pitch contour) activated by the standard T2 would also differ from the acoustic low pitch contour extracted from the deviant T3.

**Table 1:** Predictions of the MMN results based on different models

Different models	Oddball conditions (Standard/Deviant)			
	T1/T3	T3/T1	T2/T3	T3/T2
FUL	conflict	no conflict	conflict	no conflict
	MMN	reduced MMN	MMN	reduced MMN
MV	conflict	conflict	conflict	no conflict
	MMN	MMN	MMN	reduced MMN
CI	conflict	conflict	conflict	conflict
	MMN	MMN	MMN	MMN

NOTE: FUL indicates the featurally underspecified lexicon model; MV indicates the multi-variant account; CI indicates the context-based inference account. Conflict indicates mismatch between the high-level long-term phonological representation in the mental lexicon, activated by the standard and the surface form, extracted from the deviant.

Different models of lexical representation, however, would make different predictions for the reversed T3/T2 and T3/T1 conditions. Within the FUL model, T3, given its variability in context, is expected to be underspecified in the mental lexicon. Therefore, in both T3/T1 and T3/T2 conditions, no conflict (related to long-term memory representation) is expected. Previous studies demonstrated that MMN has a higher magnitude if the mapping involves a conflicting rather than a non-conflicting situation [8,49]. So, we expect to observe reduced MMNs when T3 serves as a standard, and correspondingly, asymmetrical MMNs in both T1/T3 vs. T3/T1 condition and T2/T3 vs. T3/T2 condition.

With regard to the hemisphere dominance underlying tonal processing, if indeed T3 is underspecified (FUL), we expect more left-lateralized patterns of hemisphere processing in the T1/T3 and T2/T3 conditions, given their between-category tonal processing, but not necessarily so in the T3/T1 and T3/T2 conditions, given the underspecified representation of the standard T3.

According to the MV account, multiple tonal variants can co-exist in the mental lexicon for T3; both its canonical form and its variant (with a rising pitch contour) are therefore expected to be activated by the standard T3. The latter is dissimilar to the pitch contour extracted from the deviant T1 and similar to that extracted from the deviant T2. Therefore, conflict is expected in the T3/T1 condition but not in the T3/T2 condition. Symmetrical MMNs are expected in T1/T3 vs. T3/T1 conditions while the T3/T2 condition is expected to show reduced MMNs compared to the T2/T3 condition.

Under the assumption that multiple tonal variants are stored, we expect that the T1/T3, T3/T1, and T2/T3 conditions should involve clear between-category tonal contrasts and therefore left-hemisphere dominant processing. The T3/T2 condition, however, may recruit different, right lateralized neural basis of processing, given that the standard T3 may activate T3V, which is similar to the pitch contour extracted from the deviant T2; and consequently, T3V and T2 may be processed in a way similar to within-category difference.

As for the CI account, its core essence is that the processing of speech variants is context-specific. Within this possibility, T3V is not stored in the lexical representation but computed on-line from the underlying T3 during the speech encoding process (e.g. [61–63]). Given that our study uses monosyllabic morphemes produced in isolation, it is then clear that there should be no licensing context for tonal variants. The long-term memory representation activated by the standard T3 is therefore expected to be different from both the deviant T1 and the deviant T2. Hence symmetrical MMNs are expected in the T1/T3 and T3/T1 conditions as well as in the T2/T3 and T3/T2 conditions.

This third possibility (CI) predicts more left-lateralized patterns of hemisphere processing for all conditions given that T3 is represented as its canonical tonal form, and the standard and deviant stimuli should then be consistently perceived as different lexical tones across conditions and induce between-category functional processing.

One additional issue, ancillary to our central research question but nevertheless important, can be addressed with our design. This concerns the effect of acoustic distances between different lexical tonal pairs on the neural processing of lexical tones. By comparing the MMNs elicited in T1/T3 vs. T2/T3 conditions or T3/T1 vs. T3/T2 conditions, we expect to replicate, with naturally-produced stimuli, the findings of [48] which used synthesized stimuli, on the timing and magnitude of MMN effects introduced by different acoustic distances of lexical tonal pairs.

## Method

### Ethics statement

All participants provided written informed consent in accordance with the Declaration of Helsinki. The ethics committee of the Institute of Psychology at the Chinese Academy of Sciences approved this study, its participant-recruitment procedure and its methodology.

### Participants

Twenty right-handed subjects, as measured by the Edinburgh handedness questionnaire (18–24 years old; 18 females), participated in the experiment. All of them were university students and native speakers of Mandarin dialects (from Beijing and its surrounding areas). None of them had any neurological impairment, neurological trauma, or used neuroleptics.

### Stimuli

**Fig 1:**
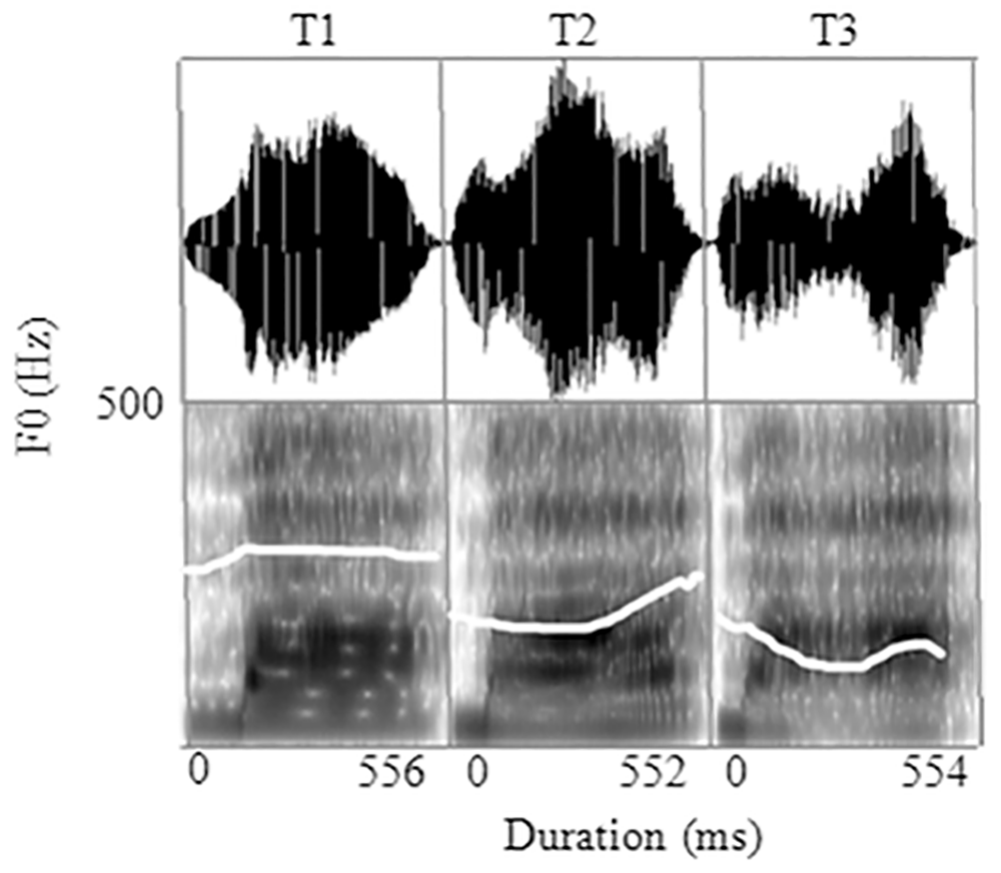
The voice spectrographs and uncorrected fundamental frequency contours (white line) for the three lexical tones: T1 (high level tone), T2 (high rising tone), and T3 (low dipping tone). (Figure created using PRAAT software: PRAAT Ver. 4.6.01, University of Amsterdam, The Netherland.)

The auditory stimuli were a set of three Mandarin Chinese words that are distinguished by lexical tones only: ma^T1^ ‘mother’ [T1], ma^T2^ ‘linen’ [T2], and ma^T3^ ‘horse’ [T3]. The words were produced by a female speaker of Standard Chinese who was born and grew up in Beijing. Tonal durations for the three naturally-elicited words ‘ma^1^’, ‘ma^2^’, and ‘ma^3^’ are 556 ms, 552 ms, and 554 ms respectively. Their voice amplitudes are 72 dB, 72 dB, and 70 dB respectively. As shown in Fig 1, T1 has a high Level *f0* contour (onset: 295 Hz; offset: 312 Hz). T2 has a high rising *f0* contour (onset: 234 Hz; offset: 281; turning point: 224 Hz around the midpoint of the tone-bearing syllable). T3 has a dipping *f0* contour (onset: 233 Hz; offset: 204 Hz; turning point: 172 Hz, slightly after the midpoint of the tone-bearing syllable).

The experiment consisted of four oddball conditions (standard/deviant, 85% /15%). In the first two oddball conditions, T3 was always used as the deviant stimuli, with the standard stimuli being T1 in one condition (T1/T3, standard/deviant) and T2 in the other condition (T2/T3). For the other two oddball conditions, the standards and deviants were reversed. One included T3 as standard and T1 as deviant (T3/T1) and the other T3 as standard with T2 as deviant (T3/T2). In addition, there were three control conditions (i.e., with just one type of stimuli): T1 (100%), T2 (100%), and T3 (100%).

### Procedure

After the electrodes were positioned, subjects were seated in an acoustically and electrically shielded room, facing a computer screen. They were instructed to watch a silent movie while listening to the auditory stimuli passively. To ensure that subjects focused their attention on the silent movie, they were informed that questions about this movie would be asked afterwards.

The whole experiment, with a total of 7 blocks, lasted about 70 minutes. For the four oddball conditions, each block consisted of a standard (p = 0.85, 567 trials) and a deviant (p = 0.15, 100 trials). For the three control conditions, the deviants in the four oddball conditions were presented alone: T1, T2, and T3 (100%, 667 trials). The inter-trial interval was 400 ms. All oddball blocks preceded the control blocks. The order of the respective blocks was counterbalanced among participants. Within each oddball block, the order of stimuli presentation was pseudo-random, i.e., with at least two standard stimuli preceding each deviant. Furthermore, every block began with an additional 10 trials, which were all standard stimuli.

### EEG acquisition

EEG was recorded (0.05–100 Hz, sampling rate 500 Hz) from 64 Ag/AgCl electrodes mounted in an elastic cap (Neuroscan Inc.), with an on-line reference to the nose. EEG and EOG data were amplified with AC amplifiers (Neuroscan). Vertical eye movements were monitored via a supra- to sub- orbital bipolar montage. A right to left canthal bipolar montage was used to monitor horizontal eye movements. All electrode impedance levels (EEG and EOG) were kept below 5 kΩ.

### ERP analysis

Data of four participants were excluded from further ERP analyses due to excessive artifacts. The remaining data from a total of 16 participants were subsequently analyzed. The first ten trials of all blocks were not included in the analysis.

The raw EEG data were first corrected for eye-blink artifacts using the ocular artifact reduction algorithm in the Neuroscan v. 4.3 software package. Then, the EEG data were filtered with a band-pass filter of 0.8–30 Hz. Subsequently, the filtered data were divided into epochs ranging from 100 ms before the onset of the words to 800 ms after the onset of the words. A time window of 100 ms preceding the onset of the words was used for baseline correction. Trials contaminated by eye movements, muscle artifacts, electrode drifting, amplifier saturation, or other artifacts were identified with semiautomatic artifact rejection (automatic criterion: signal amplitude exceeding ±75μV, followed by a manual check). Trials containing the above-mentioned artifacts were rejected (12.5% overall). Rejected trials were evenly distributed among conditions.

To guarantee that the results of our study can be directly compared to previous studies, two methods were used to calculate MMN. One is to subtract the stimuli presented in the 100% control condition from the same stimuli that were presented as deviant in the oddball condition (MMN-a) (following e.g., [48,64–66]). The other is to subtract the standard stimuli in one oddball condition from the same stimuli that were presented as deviant in the reversed oddball condition (e.g., the standard T3 in T3/T1 was subtracted from the deviant T3 in T1/T3) (MMN-b; also known as identity MMN) (following e.g., [7,59]). To match the number of deviant trials (100 trials) in the oddball condition, for MMN-a, 100 trials (which were at the same position as the deviants in the oddball sequences) in the 100% control condition were selected. For MMN-b, the standards immediately preceding and following the deviants in the oddball condition were deleted, and then, 100 trials were randomly selected from the remaining standard trials. Both MMN-a and MMN-b can therefore effectively control for acoustical differences between stimuli, as the deviant was subtracted by the same stimulus.

It may be necessary to point out that short-term habituation might influence MMN amplitude, since the deviant stimuli (e.g., T3 in T1/T3) was only presented 100 times in one block, but the same stimuli coming from the reversed oddball block (e.g., T3 in T3/T1) or the 100% control block was presented 567 or 667 times. It is important to note, however, that in the present study, we are not interested in the absolute MMN amplitude in a specific oddball condition, but interested in the relative MMN amplitude. That is, the present study hinged upon the similarity or differences between the MMNs elicited in the different oddball conditions, as described in the last part of the introduction section. The short-term habituation effect, if it exists, would be the same in the different oddball conditions, ruling out its potentially confounding effect.

**Fig 2:**
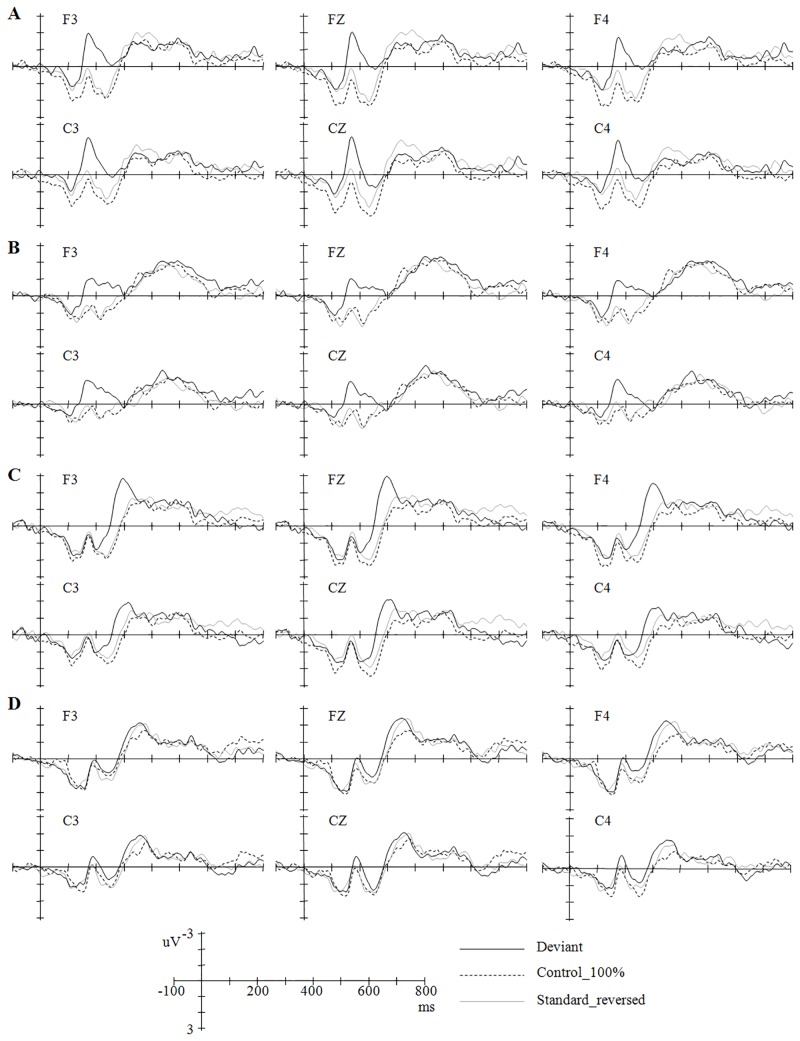
Grand-average ERPs time-locked to the deviant, standard, control stimuli in the different oddball conditions. The stimuli in A include T3 in T1/T3 (standard/deviant), T3 (100%), and T3 in T3/T1. The stimuli in B include T1 in T3/T1, T1 (100%), and T1 in T1/T3. The stimuli in C include T3 in T2/T3, T3 (100%), and T3 in T3/T2. The stimuli in D include T2 in T3/T2, T2 (100%), and T2 in T2/T3.

**Fig 3:**
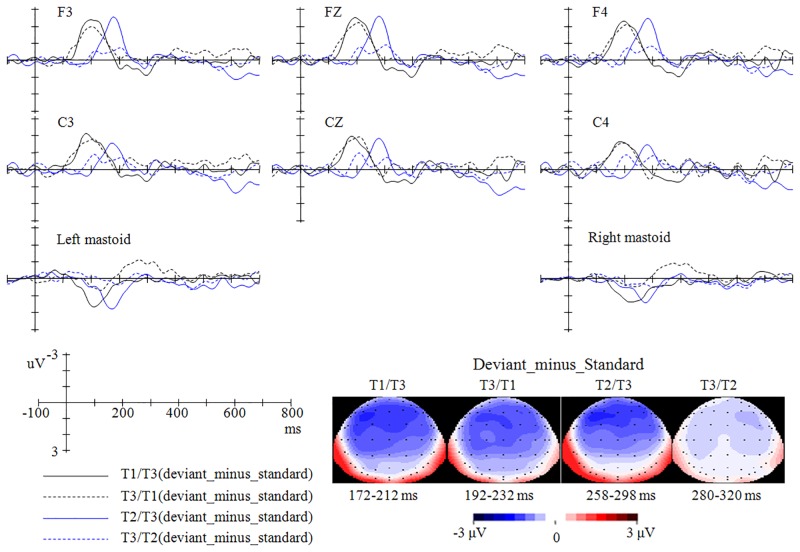
The MMN-a (difference waveforms, namely, ‘the stimuli presented as deviant in the oddball condition’-minus-‘the same stimuli presented in the 100% control condition’) in the four oddball conditions and their topographies.

**Fig 4:**
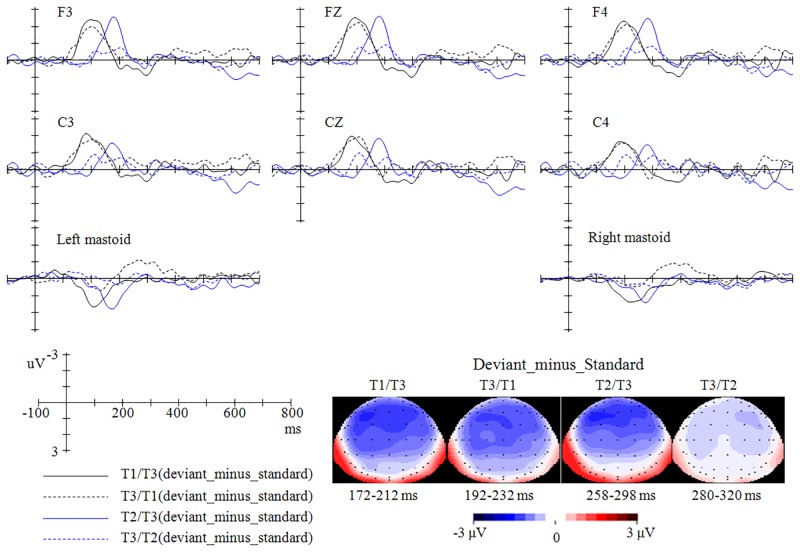
The MMN-b (difference waveforms, namely, ‘the stimuli presented as deviant in one oddball condition’-minus-‘the same stimuli presented as standard in the reversed oddball condition’) in the four oddball conditions and their topographies.

As shown in Fig 2, the deviant stimuli in the four oddball conditions all elicited larger negative deflections than the same stimuli presented in the 100% control condition or in their corresponding reversed oddball conditions. The MMNs (for both MMN-a and MMN-b) in the four conditions (T1/T3, T3/T1, T2/T3, and T3/T2) peaked after the stimulus onset around 192 ms, 212 ms, 278 ms, and 300 ms respectively (see Figs 3 and 4).

**Fig 5:**
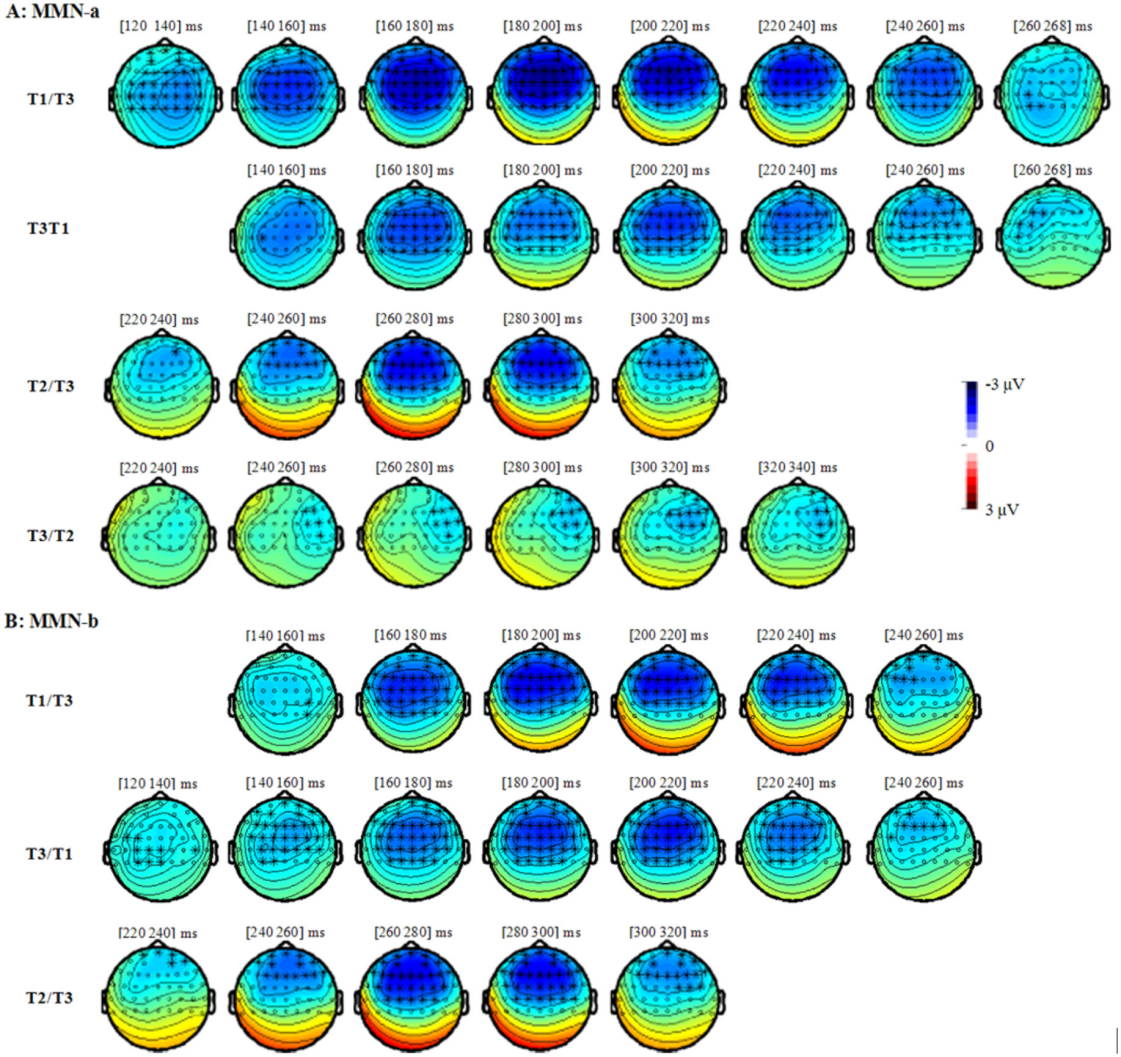
The results of the cluster-based permutation test to identify MMN (permutation test-1). The dots and the asterisks in the topography indicate the 43 frontal-central electrodes that were included in this permutation test. The asterisks indicate the electrodes over that the MMN reach significance in this permutation test. Although the cluster-based permutation tests were conducted in the step of 2 ms, the figures were shown in 20 ms step due to the limited space. Within every 20 ms, we got the similar pattern of results.

For statistical analysis, we employed the cluster-based permutation test in the Fieldtrip (http://fieldtrip.fcdonders.nl) soft-ware package [67] to examine whether MMNs were elicited by the oddball conditions and whether MMN amplitudes were significantly different across different conditions. This non-parametric statistical procedure optimally handles the multiple-comparisons problem. Frontal-central electrodes (see Fig 5) were included in this test, since auditory MMN usually has a frontal-central distribution [8,14,48]. Two kinds of permutation test were performed within 0–500 ms post-word onset in steps of 2 ms: over all of the 43 frontal-central electrodes (permutation test-1); over the right (19 electrodes) or left (19 electrodes) electrodes respectively (permutation test-2). For every data point (‘electrode by time’) of two conditions, a simple dependent-samples *t* test was performed. All adjacent data points exceeding a preset significance level (0.05%) were grouped into clusters. Cluster-level statistics were calculated by taking the sum of the *t*-values within every cluster. The significance probability of the clusters was calculated by means of the so-called Monte Carlo method with 1000 random draws.

Second, based on the results of the cluster-based permutation test (which suggest the window latencies that demonstrated the strongest MMN effect) and visual inspection of the ERP waveforms, traditional ANOVAs (analyses of variance) were conducted within the specific time windows on a selection of two midline electrodes (Fz, Cz) and four lateral electrodes (F3/F4; C3/C4). First, to estimate whether MMNs were elicited by the oddball conditions, ANOVA-1 was conducted with Condition (T1/T3, T3/T1, T2/T3, and T3/T2), Stimulus type (deviant, control), Laterality (left, midline, right), and Anteriority (frontal F3/Fz/F4, central C3/Cz/C4) as independent factors. Second, to compare the amplitude or peak latency of MMNs elicited by the four oddball conditions, ANOVA-2 was performed based on four different waveforms (T1/T3, T3/T1, T2/T3, and T3/T2), with Condition, Laterality, and Anteriority as independent factors. When the degree of freedom in the numerator was larger than one, the Greenhouse-Geisser correction was applied. Whenever applicable, Bonforroni correction was used to counteract the problem of multiple comparisons.

## Results

### The existence of MMN effects

The cluster-based permutation test over all frontal-central electrodes (permutation test-1) revealed that, for MMN-a, the MMN effect reached significance within around 100–280 ms, 140–280 ms, 240–340 ms, and 240–360 ms for T1/T3 (“deviant T3 from T1/T3” vs. “T3 from 100%”, *p* < .0001), T3/T1 (“deviant T1 from T3/T1” vs. “T1 from 100%”, *p* < .01), T2/T3 (“deviant T3 from T2/T3” vs. “T3 from 100%”, *p* < .005), and T3/T2 (“deviant T2 from T3/T2” vs. “T2 from 100%”, *p* < .05) conditions respectively. The MMNs in the T1/T3, T3/T1, and T2/T3 conditions have a broad distribution, while the MMN in the T3/T2 condition has a right-hemisphere distribution (see Fig 5A). Permutation test-2, which examines the MMNs over the left and right electrodes separately, provided further evidence that, for the T1/T3, T3/T1, and T2/T3 conditions, the MMN effects reached significance over both hemispheres; in contrast, for the T3/T2 condition, the MMN effect reached significance only over the right electrodes.

For the MMN-b, permutation test-1 revealed that, the MMN effect reached significance around 140–260 ms, 120–260 ms, and 220–320 ms for T1/T3 (“deviant T3 from T1/T3” vs. “standard T3 from T3/T1”, *p* < .0001), T3/T1 (“deviant T1 from T3/T1” vs. “standard T1 from T1/T3”, *p* < .0001), and T2/T3 (“deviant T3 from T2/T3” vs. “standard T3 from T3/T2”, *p* < .005) conditions respectively (see Fig 5B). However, the T3/T2 condition (“T2 from T3/T2” vs. “T2 from T2/T3”) did not elicit significant MMN effects in permutation test-1 or in permutation test-2. Furthermore, permutation test-2 demonstrated that, for the T1/T3, T3/T1, and T2/T3 conditions, the MMN effects reached significance over both left and right hemispheres.

Based on the results of the permutation test (for window latencies that demonstrated the strongest MMN effect) and visual inspection of the ERP waveforms, ANOVA was conducted within the window latencies of 172–212 ms, 192–232 ms, 258–298 ms, and 280–320 ms for the MMNs elicited in the T1/T3, T3/T1, T2/T3, and T3/T2 conditions respectively.

For MMN-a, the ANOVA-1 analysis revealed a significant main effect of Stimulus type (*F*_(1, 15)_ = 44.35, *p* < .0001, *d* = .75), indicating that the deviant stimuli in the oddball condition evoked a larger negative deflection (MMN) than the same stimuli in the control condition (magnitude difference: -1.85 μV). The effect of Stimulus type was qualified by a two-way Condition × Stimulus type interaction (*F*_(3, 45)_ = 6.96, *p* < .005, *d* = .32) as well as a three-way Condition × Stimulus type × Hemisphere interaction (*F*_(6, 90)_ = 2.88, *p* < .05, *d* = .16). Subsequent simple analyses showed that, for T1/T3, T3/T1, and T2/T3 conditions, the MMN effects reached significance over left, middle, and right hemispheres (T1/T3: *F*_left(1, 15)_ = 30.99, *p* < .0001, *d* = .68, *F*_middle(1, 15)_ = 35.66, *p* < .0001, *d* = .70, and *F*_right(1, 15)_ = 34.22, *p* < .0001, *d* = .69; T3/T1: *F*_left(1, 15)_ = 24.37, *p* < .0001, *d* = .62, *F*_middle(1, 15)_ = 19.05, *p* < .001, *d* = .63 and *F*_right(1, 15)_ = 11.40, *p* < .005, *d* = .43; T2/T3: *F*_left(1, 15)_ = 29.28, *p* < .0001, *d* = .66, *F*_middle(1, 15)_ = 34.97, *p* < .0001, *d* = .70, and *F*_right(1, 15)_ = 29.18, *p* < .0001, *d* = .66). However, for the T3/T2 condition, the MMN effect reached significance only over the right electrodes (*F*_(1, 15)_ = 14.08, *p* < .005, *d* = .48).

For MMN-b, the ANOVA-1 also revealed a significant main effect of Stimulus type (*F*_(1, 15)_ = 42.19, *p* < .0001, *d* = .74), suggesting that the deviant stimuli evoked a larger negative deflection (MMN) than the same stimuli presented as standard in the reversed oddball condition (magnitude difference: -1.62 μV). The two-way Condition × Stimulus type interaction (*F*_(3, 45)_ = 3.2, *p* < .05, *d* = .18), three-way Condition × Stimulus type × Anteriority interaction (*F*_(3, 45)_ = 5.15, *p* < .01, *d* = .26), and the four-way Condition × Stimulus type × Anteriority × Hemisphere interaction (*F*_(6, 90)_ = 2.60, *p* = .055, *d* = .15) all reached significance or marginal significance. Further simple analyses showed that, for the T1/T3, T3/T1, and T2/T3 conditions, the MMN effects reached significance over all of the six brain areas (T1/T3: *F*_frontal-left(1, 15)_ = 23.13, *p* < .0001, *d* = .61, *F*_frontal-middle(1, 15)_ = 24.82, *p* < .0001, *d* = .62, *F*_frontal-right(1, 15)_ = 28.63, *p* < .0001, *d* = .66, *F*_central-left(1, 15)_ = 14.33, *p* < .005, *d* = .49, *F*_central-middle(1, 15)_ = 14.81, *p* < .005, *d* = .52, *F*_central-right(1, 15)_ = 16.76, *p* < .001, *d* = .53) (T3/T1: *F*_frontal-left(1, 15)_ = 31.60, *p* < .0001, *d* = .68, *F*_frontal-middle(1, 15)_ = 40.89, *p* < .0001, *d* = .73, *F*_frontal-right(1, 15)_ = 28.72, *p* < .0001, *d* = .66, *F*_central-left(1, 15)_ = 18.46, *p* < .001, *d* = .55, *F*_central-middle(1, 15)_ = 25.98, *p* < .0001, *d* = .63, *F*_central-right(1, 15)_ = 15.86, *p* < .001, *d* = .51) (T2/T3: *F*_frontal-left(1, 15)_ = 46.54, *p* < .0001, *d* = .76, *F*_frontal-middle(1, 15)_ = 31.86, *p* < .0001, *d* = .68, *F*_frontal-right(1, 15)_ = 27.53, *p* < .0001, *d* = .65, *F*_central-left(1, 15)_ = 10.50, *p* < .005, *d* = .41, *F*_central-middle(1, 15)_ = 13.84, *p* < .005, *d* = .48, *F*_central-right(1, 15)_ = 8.93, *p* < .01, *d* = .37); however, for the T3/T2 condition, the MMN effect again reached significance only over the midline frontal (*F*_(1, 15)_ = 5.36, *p* < .05, *d* = .25) and right frontal electrodes (*F*_(1, 15)_ = 5.60, *p* < .05, *d* = .25).

In sum, both MMN-a and MMN-b showed that the deviants in the four oddball conditions all elicited a MMN effect, with the MMNs in the T1/T3, T3/T1, and T2/T3 conditions present over both left and right hemispheres while the MMN in the T3/T2 condition present only over the midline and right electrodes. Although for MMN-b, T3/T2 condition elicited no significant MMN in the cluster-based permutation test, this condition did elicit significant MMNs over the midline and right electrodes in the ANOVA analysis.

### MMN mean amplitude

**Fig 6:**
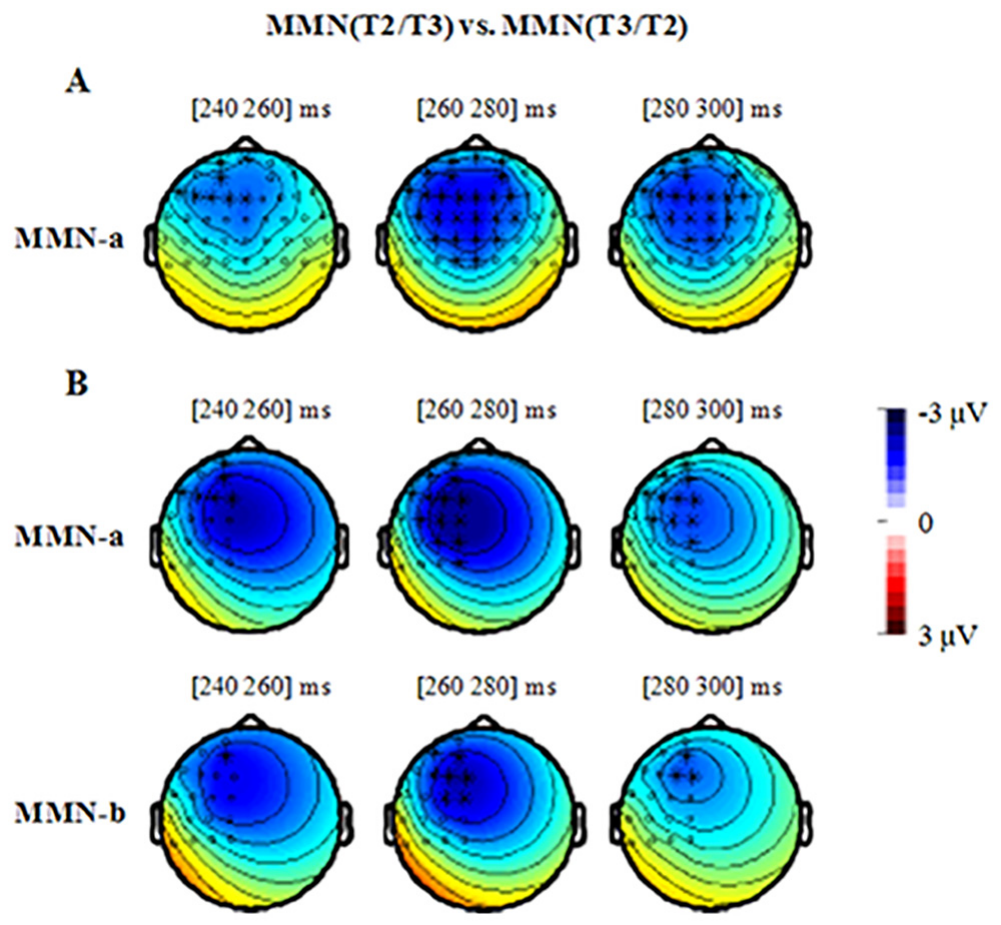
The results of the cluster-based permutation test to compare the MMNs in the different conditions. A: the permutation test over the 43 frontal-central electrodes. B: the permutation test over the 19 left electrodes. The dots and the asterisks in the topography indicate the electrodes that were included in the permutation test. The asterisks indicate the electrodes over that the difference between T2/T3 and T3/T2 condition reach significance. The only significant effect we observed was that the MMN in the T2/T3 condition was larger than that in the reversed T3/T2 condition. Although the cluster-based permutation tests were conducted in the step of 2 ms, the figures were shown in 20 ms step due to the limited space. Within every 20 ms, we got the similar pattern of results.

Similar permutation tests, but with MMN amplitude as the dependent factor, were performed to examine whether MMNs were different between every two oddball conditions. The Permutation test-1 revealed that MMN-a in the reversed T2/T3 condition was significantly larger than that in the T3/T2 condition around 240–300 ms (*p* < .05), which mainly occurred over the left hemisphere (see Fig 6A). However, the MMN-a in the T1/T3 condition was not different from that in the T3/T1 condition (*p* = .20). Furthermore, permutation test-2 showed that, for both MMN-a and MMN-b, the T2/T3 condition elicited larger MMNs than the reversed T3/T2 condition around 240–300 ms over the left hemisphere (both *ps* < .05), but not over the right hemisphere (*p* = .06 for MMN-a, *p* = .09 for MMN-b); the difference between MMNs in the T1/T3 and T3/T1 conditions did not reached significance over the left hemisphere or the right hemisphere (*ps* >.20).

For ANOVA-2, the MMN mean amplitude was computed based on the four difference waveforms (T1/T3, T3/T1, T2/T3, and T3/T2) in their corresponding latency windows (172–212 ms, 192–232 ms, 258–298 ms, and 280–320 ms respectively).

For MMN-a, ANOVA-2 resulted in a significant main effect of Condition (*F*_(3, 45)_ = 6.96, *p* < .005, *d* = .32) and a significant two-way Condition × Hemisphere interaction (*F*_(6, 90)_ = 2.88, *p* < .05, *d* = .16). The simple main effect of Condition reached significance over the left hemisphere (*F*_(3, 45)_ = 8.08, *p* < .001, *d* = .35), midline electrodes (*F*_(3, 45)_ = 7.47, *p* < .005, *d* = .33), and right hemisphere (*F*_(3, 48)_ = 4.39, *p* < .05, *d* = .23). Follow-up pairwise comparisons revealed that the MMN amplitude in the T1/T3 condition was not different from that in the reversed T3/T1 condition (*p* = 0.30; *p* = 0.08; *p* = 0.20 for left, midline, and right areas respectively); in contrast, the MMN amplitude in T2/T3 condition was significantly larger than that in the reversed T3/T2 condition over the left and midline electrodes (left: *p* < .05, magnitude difference: -1.75 μV; Middle: *p* < .05, magnitude difference: -1.76 μV). In addition, the other pairwise comparisons did not reach significance.

For MMN-b, ANOVA-2 resulted in a significant main effect of Condition (*F*_(3, 45)_ = 3.20, *p* < .05, *d* = .18) and a significant two-way Condition × Anteriority interaction (*F*_(3, 45)_ = 4.82, *p* < .01, *d* = .23) and a significant three-way Condition × Anteriority × Hemisphere interaction (*F*_(6, 90)_ = 2.60, *p* = .055, *d* = .15). The follow-up pairwise comparisons over the six brain areas revealed that the MMN amplitude in the T2/T3 condition was larger than that in the reversed T3/T2 condition only over the left frontal electrode (*p* < .05; magnitude difference: -1.71 μV). The other comparisons didn’t reach significance.

Thus, for both MMN-a and MMN-b, the MMN amplitude in the T1/T3 condition was not different from that in the reversed T3/T1 condition. However, the MMN amplitude in T2/T3 condition was larger than that in the reversed T3/T2 condition over the left hemisphere. In addition, there was no significant difference between the MMNs in the T1–T3 pair (larger acoustic distance) and T2–T3 pair (smaller acoustic distance) conditions.

### MMN peak latency

Finally, we examined the timing characteristics of MMNs evoked in the four oddball conditions. Peak latency of the negative deflection in the four MMNs (T1/T3, T3/T1, T2/T3, and T3/T2,) was measured by determining individual peak latencies in the following latency windows: 120–260 ms, 120–260 ms, 220–350 ms, and 220–350 ms after the onset of the deviant stimulus respectively. This time-window was set after visual inspection of the window latencies for the corresponding MMNs in the present result.

For MMN-a, ANOVA-2 with peak latency as dependent factor resulted in a significant main effect of Condition (*F*_(3, 45)_ = 55.04, *p* < .0001, *d* = .79). The subsequent pairwise comparisons revealed that neither the difference between the T1/T3 and T3/T1 conditions (p = 1.000) nor the difference between the T2/T3 and T3/T2 conditions (p = .542) reached significance. However, MMNs in the T1/T3 condition (195 ms) peaked earlier than that in both the T2/T3 condition (273 ms) (p < .0001) and the T3/T2 condition (290 ms) (p < .0001); MMNs in the T3/T1 contrast condition (203 ms) also peaked earlier than that in both the T3/T2 (p < .0001) and the T2/T3 conditions (p < .0001).

For MMN-b, ANOVA-2 with peak latency as dependent factor also resulted in a significant main effect of Condition (*F*_(3, 45)_ = 76.997, *p* < .0001, *d* = .84). The subsequent pairwise comparisons also showed that neither the difference between the T1/T3 and the reversed T3/T1 (p = 1.000) conditions nor the difference between the T2/T3 and the reversed T3/T2 (p = .864) conditions reached significance. However, the MMN effects in the T1/T3 condition (198 ms) peaked earlier than that in the T2/T3 (278 ms) condition (p < .0001) and that in the T3/T2 (291 ms) condition (p < .0001); the MMN effects in the T3/T1 condition (204 ms) also peaked earlier than that in the T3/T2 (p < .0001) and the T2/T3 conditions (p < .0001).

To summarize, for MMN peak latency, neither the difference between T1/T3 and the reversed T3/T1 nor the difference between T2/T3 and the reversed T3/T2 reached significance. However, MMNs elicited in conditions with big acoustic differences (i.e. T1/T3 and T3/T1) peaked earlier than that with relatively smaller acoustic differences (i.e. T2/T3 and T3/T2).

## Discussion

With a passive oddball MMN paradigm, the present study examined the representation and neural processing of lexical tones in Standard Chinese. A reversed design was adopted to control the standard-deviant acoustic contrast so as to tap specifically into the possible effects of allotonic variation on tonal representation and neural processing in general. Significant MMN effects were found for the T1/T3, T3/T1, and T2/T3 conditions over both the left and right hemispheres. For the T3/T2 condition, however, the MMN responses were much more reduced and had a right hemisphere lateralization. More importantly, the comparison of these MMN effects revealed symmetrical MMN effects for the T1–T3 pair but asymmetrical MMNs for the T2–T3 pair.

Before we consider these results in more detail, it is important to note that different acoustic distances between lexical tonal pairs (i.e. T1–T3 vs. T2–T3) did introduce different MMN effects. As introduced earlier, in Standard Chinese, the pitch contour of T3 is acoustically more similar to T2 than to T1. Our results showed that the MMNs evoked by the tonal pair T1–T3 peaked earlier than the MMNs evoked by the tonal pair T2–T3 where there is smaller acoustic difference and later identification point. The present study thus replicated the results reported in [48] which showed earlier-peaked MMNs in the T1/T3 condition compared to the T2/T3 condition. This also echoes the general observation that MMN responses originate from the magnitude of acoustic contrasts between the standard and deviant stimuli [68].

Note that Chandrasekaran et al. [48] also found a significantly larger amplitude in the T1/T3 condition than that in the T2/T3 condition. This, however, was not replicated in our study. Note that their study used synthesized pitch contours while our stimuli were produced naturally by a native speaker of Standard Chinese. The lack of amplitude difference in our study is likely due to the richer acoustic cues in the naturally produced lexical tones which can probably cue more effectively the lexical tonal contrasts, and consequently led to greater MMN effects in our T2/T3 condition. This enhanced MMN effects in the T2/T3 condition in our study, however, stands in stark contrast to the weakened and non-significant MMN effects in the T3/T2 condition.

What cries for explanation is thus the two intriguing patterns of asymmetry that have emerged in our study. First, the T2–T3 pair showed different MMN effects in the T3/T2 vs. T2/T3 condition, in contrast to the comparable MMN effects for the tonal pair T1–T3 (which have rather distinct pitch contours in all tonal contexts). Second, the MMN effects in the T3/T2 condition were mainly observed in the right hemisphere, in contrast to the broad (both right and left hemispheres) MMN effects observed in the T2/T3 condition, as well as in the T1/T3 and T3/T1 conditions. The present results thus demonstrated that with the same amount of acoustic distance between the standard and deviant auditory stimuli, T1/T3 elicited symmetrical MMNs as the reversed T3/T1 condition. The T2–T3 pair, however, despite that the holistic acoustic distance in the T2/T3 condition remains the same as those in the T3/T2 condition, showed significantly different MMN effects not only in terms of MMN amplitude, but also in terms of their scalp distribution.

These differences clearly cannot be explained by mere acoustic features, especially given our standard-deviant reverse design. In the following, we will argue that such asymmetries shed light on the effect of allophonic tonal variants on the representation (Section 4.1) and neural processing (Section 4.2) of lexical tones in general. Implications of these results on proper modeling of pronunciation variation in speech processing will be taken up and discussed in Section 4.3.

### The effect of allophonic variation on lexical tone representation

The central goal of the present study was to investigate the effect of allophonic lexical tone variants on the representation of lexical tones in general. To recapitulate, in Standard Chinese, T3 has an allophonic tone sandhi variant T3V, which surfaces only in specific tonal context (i.e. in a T3T3 sequence). T3V has a rising pitch contour, which is acoustically similar to (and often perceptually undistinguishable from) the rising pitch contour of the lexical T2. T3 and T3V, however, are always quite distinct from the lexical T1, which has a level pitch contour (Fig 1). Note that in our experiment, listeners were only exposed to T3 produced in isolation, which are categorically different from both T1 and T2 with near ceiling-level accuracy in tonal identification [60]. We were interested in whether, without the specific tonal sandhi context (i.e. T3 produced in isolation), T3V can be activated, which should constitute as convincing evidence for the effect of tone sandhi variant (T3V) on the memory representation of lexical T3 in general.

Results of the oddball paradigm showed significant and widely distributed MMN effects in the T1/T3, T3/T1, and T2/T3 conditions across the left, middle, and right electrodes, as one would have expected for lexical tone processing. In the T3/T2 condition, however, we observed much more reduced and restricted MMN effects, which was only significant in the right hemisphere. Further comparisons showed that for the tonal pair T1–T3, MMN in the T1/T3 condition did not show any significant difference from that in the reversed T3/T1 condition both in terms of MMN amplitude and peak alignment; however, for the tonal pair T2–T3, MMN was significantly larger when T2 was the standard and T3 the deviant (T2/T3), as compared to the reversed T3/T2 condition.

One may be tempted to attribute the asymmetrical MMNs to the different directions of pitch change between standard and deviant stimuli in the four oddball blocks. Gomes et al. [69], however, showed that low-to-high and high-to-low pitch changes of pure tones do not introduce significant differences in the magnitude and scalp distribution of MMNs, suggesting that pitch direction change should not have brought the observed asymmetry. Furthermore, we know that T1 has a high level pitch contour, T2 a high rising contour, and T3 a low rising contour. As is clear from Fig 1, in the T2–T3 pair, their difference only lies in the magnitude of pitch falling from high to low. In the T1–T3 pair, however, the pitch falls from high to low in T1/T3 and rises from mid to high in T3/T1. If indeed pitch direction change could have contributed to different MMN effects, we should have observed greater asymmetry of MMNs in the T1–T3 pair, and not in the T2–T3 pair. Taken together, the pitch-change direction account seems untenable for our observed asymmetry.

A related alternative is that the asymmetrical MMNs are due to other acoustic factors such as spectral and temporal differences, or frequency differences of the stimuli[50]. We think this is also highly unlikely. First, in the present study, T1 and T2 have the same intensity (72 dB). Asymmetric MMNs were observed in the T3–T2 pair but not in the T3–T1 pair. Furthermore, the durational differences in the two tonal pairs were both about 2 ms (T1: 556 ms; T2: 552 ms; and T3: 554 ms), which makes it doubtful to have induced the observed MMN asymmetry. Lexical/tonal frequency distribution patterns also raise further doubts to the third possibility. We know that ma^T3^ (0.02663) has a higher frequency than both ma^T1^ (0.00825) and ma^T2^ (0.00438). Furthermore, T3 is known as the least frequent tone among the four lexical tones. According to *Junda Chinese Text Computing*, 16% of the 9933 unique characters in modern Chinese (listed on the site) have T3 but the percentages of T1 and T2 characters are higher (25% and 26% respectively). We know that words with higher frequency occurrence in a language show a more pronounced and earlier lexical MMN response than its low-frequency counterpart [59]. Regardless of whether and which of these two types of frequency may have differential MMN effects, the fact that asymmetrical MMNs were observed only in the T3–T2 pair renders versions of frequency account implausible.

A third possibility is to relate this asymmetry to the more general mechanisms of asymmetrical perception reported in an auditory domain, such as infant vowel perception [70,71], in a visual domain, such as color perception [72], as well as in other domains, such as abstract concept categorization [73], which all show that perceptual asymmetries exist given different orders of presentation for the same stimulus pair. Such perceptual asymmetries have been attributed to the unequal perceptual saliency of the stimuli resulting from, for example, their different frequencies, distributional patterns, or familiarity, with the relatively more frequent, familiar, or more peripheral ones (e.g. in the case of vowel) typically acting as the perceptual magnet or default and therefore inducing a more discriminable ‘pop out’ effect. Following the line, one may argue then that T3 is the default lexical tone in the language, which gives rise to the perceptual saliency when T2 acts as the standard in the oddball paradigm, introducing stronger MMN effects in the T2/T3 condition than that in the T3/T2 condition. The question left unanswered, however, is the symmetrical MMN effects in the T3/T1 vs. T1/T3 conditions.

We propose that such an asymmetry can be better explained by taking into consideration the possible activation of T3V even though listeners in our experiment were exposed only to the canonical T3 production, without the presence of the T3 allophonic context. Specifically, when T3 serves as the standard, its long-term memory representation may be activated, which includes not only the canonical low tone (T3) representation but also its rising sandhi variant (T3V) representation, which has a similar rising pitch contour as T2. Thus, when listeners encountered the deviant T2, there was little conflict between the activated phonological representations (i.e. T3 and T3V) and the acoustic parameters extracted from the deviant T2. This then accounts for the weakened MMN effects in the T3/T2 condition. When T3 is the deviant and T2 standard (T2/T3), however, the pitch contour extracted from the deviant T3 (with a low pitch contour) mismatches the perceptual long-term representation activated by the standard T2 (i.e. a lexical rising tone). This contrast then gave rise to the observed asymmetrical MMNs between T2 and T3.

This account is also compatible with results from behavioral production studies on the phonological encoding of lexical tone and tonal variants in Standard Chinese with the implicit priming paradigm [74] and the picture-word interference paradigm [75], both of which suggest the co-storage of T3 and T3V in the mental lexicon. Further evidence on representation of multiple tonal variants comes from [76] which investigated the lexical access of a type of tonal variability in Jinan Mandarin − lexically non-contrastive in specific words but potentially contrastive in other words. Results of their auditory lexical decision experiment in a medium-term auditory priming paradigm showed that lexically non-contrastive word-specific tonal variability induced facilitation, in contrast to the inhibition effect found for lexically-contrastive primes, which suggests storage of word-specific multiple tonal variants. As we will show below, such a view of the long-term memory representation of T3 together with its sandhi variant T3V also helps to understand the neural basis of lexical tonal and tonal variant processing we have observed in the study.

### The effect of allophonic variation on the neural processing of lexical tones

Moving onto the effect of allophonic variation on the neural processing of lexical tones in general, it might be helpful to recapitulate that the existing literature suggests that the left hemisphere is more sensitive to between-category functional processing of lexical tones [77,78] while the right hemisphere is more sensitive to low-level acoustic processing of within-category tonal differences [42–44,79,80].

In the present study, the MMNs elicited in the four oddball conditions suggest asymmetrical patterns of hemisphere lateralization. Specifically, the deviant in the T1/T3, T2/T3, and T3/T1 conditions all elicited bilateral MMNs. Moreover, relative to the reversed T3/T2 condition, the MMN in the T2/T3 condition was enhanced only over the left hemisphere. In other words, the MMNs elicited in the T1/T3, T2/T3, and T3/T1 conditions showed the same pattern of left-hemisphere dominant lateralization with an overall more global MMN distribution (extending over to the midline and right hemisphere). This suggests that in these three conditions, the standard and deviant stimuli were perceived as categorically different lexical tones and correspondingly induced between-category functional processing of the lexical tones.

In the T3/T2 condition, however, the MMNs were more lateralized to the right hemisphere with no significant neural signatures of processing at all in the left-hemisphere. The results thus point to the possibility that both the lexical low tone T3 and its rising variant T3V were activated by the standard T3, with the latter having a similar acoustic distribution as the deviant T2. T3 as the standard, with T2 as the deviant, is then likely to be processed by the native listeners as having only acoustic difference, similar to within-category tonal processing. Consequently, the tonal processing in the T3/T2 condition showed a rightward scalp distribution, different from the other three oddball conditions.

It is worth noting that in the present study, the majority of the participants were females. One may question whether the observed patterns of lateralization can be due to just gender difference in neural processing. Previous studies have showed differential degree of hemisphere specialization in the MMNs (e.g. [81,82]). Ikezawa and colleagues [81] found that although for low-level pure-tone MMNs, there is no significant gender differences in hemispheric lateralization, males (relative to females) exhibited greater left-hemisphere lateralization with relatively high-level phonetic MMNs. In line with Ikezawa and colleagues’ results, a recent study on white matter microstructure also found that males have increased language left-lateralization, as indicated by higher FA (fractional anisotropy) in the left superior longitudinal fasciculus, and females have more efficient inter-hemispheric transfer, as indicated by higher FA in corpus callosum [82]. These findings predict that if more male listeners participated in the present study, the MMNs elicited in the three conditions T1/T3, T3/T1, T2/T3 would show increased left-hemisphere lateralization as compared to the present results, since the three conditions involved relatively high-level functional or linguistic difference; the MMN elicited in the T3/T2 condition would show a similar pattern of results as the present ones, since this condition mainly involve low-level acoustic difference. Therefore, it is less likely that the hemispheric lateralization observed in the present study can be reduced to gender-specific differences. However, since the present study was not designed specially to examine the gender difference and did not directly manipulate the high-level lexical-semantic variation of the lexical tone, the gender or acoustic/functional effects on the hemisphere lateralization of tonal variation processing needs to be further studied in the future.

To summarize, although ERP responses recorded on the scalp are not always induced by a definitely stronger left/right lateralization in the cortex, the more rightward scalp distribution of MMNs in the T3/T2 condition, as compared to the bilateral scalp distribution in the other three oddball conditions, is consistent with the neural basis of cross-category vs. within-category lexical tone processing. This, in turn, provides further evidence for the hemispheric preference and their functional roles as related to high-level cognitive and lower-level sensory properties of lexical tone processing, along the line of the model put forward by Gandour and his colleagues [40,83,84].

### The three pronunciation variation models

Results of this study provide a new window for evaluating models of the representation and processing of pronunciation variation at the supra-segmental level. Following the FUL model [5–8], we would assume that lexical items are associated with one highly abstract phonological representation in the mental lexicon, where no detailed or predictable variation is stored in the mental representation. Following the MV account, we expected that multiple tonal variants can be jointly stored in the mental lexicon [17–20]. The CI account, however, assumes a single abstract lexical representation and the processing of tonal variants is context-specific (e.g. [21–24]) so that tonal variants should be only inferred given its proper context. They make different predictions for the MMN effects in the four oddball conditions as laid out in Table 1.

Our results showed an asymmetrical MMN effect in the processing of the T2–T3 pair but no difference in the T1–T3 pair. This counters the prediction of both the FUL account (which predicts asymmetrical MMNs for both pairs when T3 serves as the standard) and the CI account (which expects no asymmetry in either pair). On the other hand, our results fit well with the MV account, granted that the standard (i.e. frequently presented) T3 indeed activates the memory representations of both its canonical low tone and its rising sandhi variant (which is acoustically similar to T2), thereby inducing asymmetrical MMNs in the T2–T3 pair but not in the T1–T3 pair.

Earlier studies such as [8,14] have lent convincing support to the FUL account with evidence from underspecified featural representation. The difference between the current study on tonal variants and the earlier studies might be related to the different types of stimuli used. The earlier studies focused on featural specification in relation to segmental variation, such as the coronal place of articulation. The present study, however, investigated allophonic variation at the super-segmental level, namely, lexical tone variants. In Standard Chinese, there are only four lexical tones. Tones therefore play a very important role in distinguishing lexical meaning. Our results, in which the repeated presentation of a lexical tone activated its multiple allophonic variants, are hard to be reconciled by simply assuming T3 as the default tonal category in the language which leads to asymmetry in processing. Instead, the present study squares much better with the possibility that multiple tonal variants of a lexical item are jointly stored in the mental lexicon, leading to processing asymmetry that arises from acoustic similarities of the co-activated representations.

Our results also suggest that the activation of multiple tonal representations can be free of context. In Standard Chinese, allophonic tonal variation mainly depends on lexical tonal context. For example, only when immediately followed by another T3 in the connected speech, is the Low dipping tone (T3) realized with a rising pitch contour. In the present study, all stimuli consisted of only monosyllabic morphemes produced in isolation and were presented with an inter-trial interval of 400 ms, which, to the native ears, indisputably gives rise to the perception of two separate *ma* ^*T3*^ morphemes produced in isolation. It is also important to note that the integration of two occurrences of *ma* ^*T3*^ would result in a nonsense sequence (i.e. ma^T3^ma^T3^ which literally means ‘horse horse’). While further experimentation may be beneficial to exclude the possible phantom T3 sandhi context in a sequence of standard T3s, we feel it is on the safe side to conclude that no appropriate phonological context is licensed for tonal alternation to argue for the context-dependent inference (CI) account, where phonological variation is inferred via the presence of variation-licensing context [21–23]. This view, however, does not deny the possible role that licensing context and active inference processes can play in the perceptual treatment of pronunciation variation in speech.

## Conclusions

In conclusion, the present study used the reversed MMN paradigm to investigate the effect of lexical tonal variants on the representation and neural processing of tones in general. The current results suggest the activation of the memory representations of both canonical lexical tones and their allophonic tonal variants, even when free of allophonic tonal context. Our results provide supporting evidence for the storage of tonal variants in the mental lexicon, compatible with the multiple variant representation account. The activation of the allophonic tonal variants can lead to dominant right-hemisphere processing of lexical tones, which are otherwise categorically processed via recruiting both left and right hemispheres.

Although tonal variability is ubiquitous in speech, very little is known about their effects on the representation and processing lexical tones in general. Few neurophysiological studies examining tonal variants exist (but see [63,85]on the neural encoding of tonal variants in speech production). Furthermore, none uses the oddball paradigm with MMN effects, which is known as an effective tool to tap into the early neural processing of linguistic representations. Future studies are necessary to further understand the internal structure of lexical representations in particular with regard to allophonic tonal variants in a diverse range of languages with different patterns of tonal variation.
